# The Role of the P2X_7_ Receptor in Infectious Diseases

**DOI:** 10.1371/journal.ppat.1002212

**Published:** 2011-11-10

**Authors:** Catherine M. Miller, Nicola R. Boulter, Stephen J. Fuller, Alana M. Zakrzewski, Michael P. Lees, Bernadette M. Saunders, James S. Wiley, Nicholas C. Smith

**Affiliations:** 1 Institute for the Biotechnology of Infectious Diseases, University of Technology, Sydney, Broadway, New South Wales, Australia; 2 Nepean Clinical School, Nepean Hospital, The University of Sydney, Penrith, New South Wales, Australia; 3 Centenary Institute of Cancer Medicine & Cell Biology, The University of Sydney, Camperdown, New South Wales, Australia; 4 Florey Neuroscience Institute, The University of Melbourne, Parkville, Victoria, Australia; 5 Queensland Tropical Health Alliance, Faculty of Medicine, Health and Molecular Sciences, James Cook University, Smithfield, Cairns, Queensland, Australia; University of California San Diego, United States of America

## Abstract

ATP is an extracellular signal for the immune system, particularly during an inflammatory response. It is sensed by the P2X_7_ receptor, the expression of which is upregulated by pro-inflammatory cytokines. Activation of the P2X_7_ receptor opens a cation-specific channel that alters the ionic environment of the cell, activating several pathways, including (i) the inflammasome, leading to production of IL-1β and IL-18; (ii) the stress-activated protein kinase pathway, resulting in apoptosis; (iii) the mitogen-activated protein kinase pathway, leading to generation of reactive oxygen and nitrogen intermediates; and (iv) phospholipase D, stimulating phagosome-lysosome fusion. The P2X_7_ receptor can initiate host mechanisms to remove pathogens, most particularly those that parasitise macrophages. At the same time, the P2X_7_ receptor may be subverted by pathogens to modulate host responses. Moreover, recent genetic studies have demonstrated significant associations between susceptibility or resistance to parasites and bacteria, and loss-of-function or gain-of-function polymorphisms in the P2X_7_ receptor, underscoring its importance in infectious disease.

## Introduction

In addition to its role in cellular metabolism, the purine nucleotide ATP acts as an important extracellular messenger in a range of physiological processes, including synaptic transmissions, taste, bone formation/resorption, male fertility, blood pressure regulation, and inflammation [1]–[3]. Its effects are mediated through activation of purinergic receptors such as the P1 adenosine and the P2 nucleotide receptors [4]. Purinergic receptors are found on all types of cells in mammalian tissues, with many cells expressing multiple P1 and P2 subtypes [5].

P2 receptors are classified into two subfamilies—the P2X ligand-gated ion channels and the P2Y G protein–coupled receptors [4]. To date, seven P2X subunits (P2X_1_–P2X_7_) and eight P2Y subunits (P2Y_1_, P2Y_2_, P2Y_4_, P2Y_6_, P2Y_11_, P2Y_12_, P2Y_13_, P2Y_14_) have been identified [5]. P2X receptors are fast acting and open a cation-selective channel within milliseconds of ATP binding, whereas P2Y receptors are slower acting because their activation proceeds through second-messenger pathways, in the form of G proteins. Most P2X receptors have a low affinity for ATP, usually within the µM–mM range, while P2Y receptors are responsive to nM concentrations of ATP [6].

## The P2X Family

There are seven mammalian P2X subunits that assemble as homo- or heterotrimers to form functional receptors [7]. Each subunit consists of intracellular N and C termini and two membrane-spanning segments, separated by an extracellular loop containing ten conserved cysteine residues, thought to form disulfide bonds, and lysine and phenylalanine residues involved in activation by ATP [7]. The C termini of the various P2X subunits vary in length from 25 amino acids in the P2X_6_ receptor to 240 amino acids in the P2X_7_ receptor and are associated with the functional properties specific to each receptor [2], [8]. Each subunit is able to bind a molecule of ATP although the sensitivity to ATP binding varies widely within the family, with the P2X_1_ receptor requiring nM levels for activation, whereas the P2X_7_ receptor requires mM concentrations [3]. Brief exposure of all P2X receptors to ATP opens up a channel that renders the cell permeable to Na^+^, K^+^, and Ca^2+^, causing an increase in intracellular Ca^2+^ and Na^+^ concentrations and a decrease in intracellular K^+^. This leads to depolarisation of the cell membrane and initiation of downstream Ca^2+^ signalling pathways [3]. Prolonged exposure of the P2X_1_ and P2X_3_ receptors to ATP results in desensitisation and closure of the pore; however, prolonged exposure of the P2X_2_, P2X_4_, P2X_5_, and P2X_7_ receptors to ATP results in sustained membrane depolarisation and the opening of large transmembrane pores [2] that are permeable to hydrophilic molecules between 314 Da and 900 Da, depending on the cell type studied [9], [10].

## The P2X_7_ Receptor

The P2X_7_ receptor is highly expressed by cells of the haemopoietic lineage and can mediate cell death, killing of infectious organisms, and regulation of the inflammatory response [7], [11], [12]. The receptor is constitutively expressed. Under normal physiological conditions, activity of the receptor is kept at a low level by the extracellular concentration of divalent cations such as Ca^2+^ and Mg^2+^, which appear to alter the affinity of ATP binding in an allosteric manner, and this is believed to prevent unnecessary cell permeability and pore formation [7], [13]. Under pathophysiological conditions, for example at sites of inflammation or infection, expression is up-regulated by inflammatory cytokines [14]. Extracellular concentrations of Ca^2+^ and Mg^2+^ decrease as a consequence of dead and damaged cells releasing their cytosolic contents. This “dilution”, combined with an increase in ATP (also released from lysing cells), enhances P2X_7_ receptor activation [7], [13].

**Figure 1:**
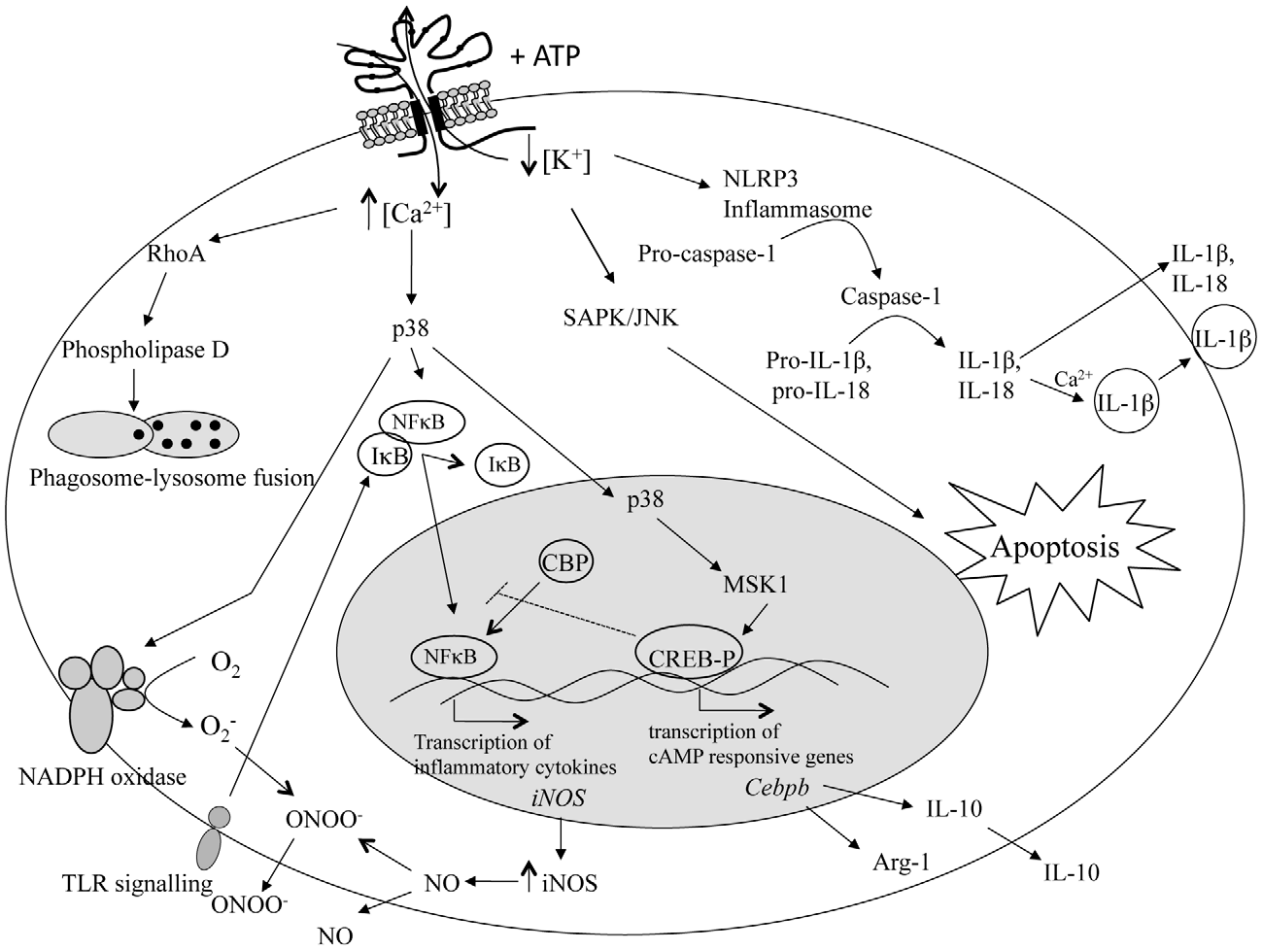
Intracellular pathways in immune cells stimulated by P2X_7_ receptor activation. Activation of the P2X_7_ receptor with extracellular ATP opens a cation-specific ion channel that results in the influx of Ca^2+^ and Na^+^ and the efflux of K^+^. Prolonged exposure to ATP creates a pore in the cell membrane that further increases the intracellular Ca^2+^ concentration as well as allowing passage of larger molecules. This alteration in the ionic environment of the cell triggers a number of cellular pathways. Efflux of K^+^ stimulates the formation of the inflammasome, resulting in the activation of caspase-1. Caspase-1 then cleaves pro-IL-1β and pro-IL-18 to produce IL-1β and IL-18, which are then secreted from the cell as part of the inflammatory response. The efflux of K^+^ and influx of Na^+^ also activates the stress-activated protein kinase (SAPK)/c-Jun N-terminal kinases (JNK) pathway, resulting in the induction of apoptosis. The influx of Ca^2+^ activates phospholipase D via RhoA, leading to phagosome/lysosome fusion and the killing of intracellular pathogens. Influx of Ca^2+^ can also activate the mitogen-activated protein kinase p38, stimulating a number of downstream effects. Phosphorylation of p38 leads to the assembly of NADPH oxidase at the plasma membrane, and the subsequent production of superoxide (O_2_^−^), enhances nuclear factor kappa B (NFκB) activation via toll-like receptor (TLR) signalling and the subsequent transcription of inducible nitric oxide synthase (iNOS) and production of nitric oxide (NO), as well as production of tumour necrosis factor (TNF) and IL-6. It can also lead to the phosphorylation of cAMP response elements binding protein (CREB) via mitogen- and stress-activated kinase 1 (MSK1). Phosphorylated CREB (CREB-P) sequesters CREB binding protein (CBP), a co-transcription factor required for NFκB-mediated gene transcription, and inhibits transcription of NFκB-controlled genes. Phosphorylated CREB/CBP also stimulates the production of cAMP-responsive genes such as *Cebpb* that act to modulate the inflammatory response through the production of arginase-1 (Arg-1) and IL-10.

The alteration in the ionic environment of the cell, resulting from P2X_7_ receptor activation, triggers a number of cellular pathways, depending on cell type (Figure 1), including: (i) the inflammasome, leading to production of IL-1β and IL-18 [15]–[17]; (ii) the stress-activated protein kinase pathway, resulting in apoptosis [18]; (iii) the mitogen-activated protein kinase pathway, leading to generation of reactive oxygen and nitrogen intermediates [11], [19]–[21]; and (iv) phospholipase D, stimulating phagosome–lysosome fusion [22]. The involvement of the P2X_7_ receptor in these pathways suggests that it functions as a major regulator of inflammation. Indeed, absence of the P2X_7_ receptor alters immune cell function with the effect seen dependent on context. For example, in a murine model of arthritis, P2X_7_ receptor–deficient animals showed a reduction in the incidence and severity of the disease compared with wild-type animals [23]; however, in a study of autoimmune encephalomyelitis, P2X_7_ receptor–deficient animals had exacerbated neuroinflammation compared with wild-type animals [24]. Moreover, a number of change-of-function polymorphisms, most of which render the receptor inactive or with reduced function, have also been noted in the human population (Table 1) [25]–[32], and genetic association studies have uncovered links between some of these polymorphisms and resistance/susceptibility to mood disorders, bone diseases, and, most particularly, infectious disease [26]. It should be noted, however, that immune cells also express P2Y receptors, such as P2Y_2_, which are also activated by inflammatory cytokines and, so, may also contribute to the regulation of inflammation [5].

**Table 1:** Single nucleotide polymorphisms identified in the human P2X_7_ receptor.

*P2X7R* Gene Polymorphism (Nucleotide Position and Base Change)	Amino Acid Change	Location in Receptor	Effect on Receptor Function	Disease Association	dbSNP ID
151+1 g>t	Produces null allele	Exon1/intron1 boundary	Loss of function – nonsense-mediated mRNA decay [25], [26]	None known	rs35933842
253 T>C	Val-76>Ala	Extracellular loop	Loss of function – partial reduction in pore formation [27]	None known	rs1752809
474 G>A	Gly-150>Arg	Extracellular loop	Loss of function – disrupted protein folding, no pore formation [27], [28]	None known	rs28360447
489 C>T	His-155>Tyr	Extracellular loop	Gain of function – enhanced pore formation and Ca^2+^ influx [29]	None known	rs208294
835 G>A	His-270>Arg	Extracellular loop	Gain of function – enhanced pore formation [27]	None known	rs7958311
853 G>A	Arg-276>His	Extracellular loop	Loss of function – no pore formation [27]	None known	rs7958316
946 G>A	Arg-307>Gln	ATP binding site	Loss of function – no channel or pore formation, loss of phospholipase D activity [30]	None known	rs28360457
1068 G>A	Ala-348>Thr	Cytoplasmic tail	Gain of function –enhanced pore formation and IL-1β secretion [27]	None known	rs1718119
1096 C>G	Thr-357>Ser	Cytoplasmic tail	Loss of function – partial reduction in channel and pore formation [25]	Impaired mycobacterial killing	rs2230911
1405 A>G	Glu-460>Arg	Cytoplasmic tail	Loss of function – partial reduction in pore formation [26], [27]	Bipolar disorder, major depressive disorder	rs2230912
1513 A>C	Glu-496>Ala	Cytoplasmic tail	Loss of function – suboptimal receptor assembly affecting pore formation [28], [31]	Susceptibility to reactivating tuberculosis; chronic lymphocytic leukaemia	rs3751143
1729 T>A	Ile-568>Asn	Cytoplasmic tail	Loss of function – prevention of receptor trafficking and surface expression [32]	None known	rs1653624

## The P2X_7_ Receptor and Intracellular Bacteria

Mycobacteria, such as *Mycobacterium tuberculosis*, the causative agent of human tuberculosis, are able to survive and replicate in phagosomes within macrophages by inhibiting phagosomal fusion with lysosomes [33]. Treatment of infected macrophages with ATP, however, can overcome the phagosome–lysosome fusion block, leading to the killing of intracellular bacilli [34]. The process appears to be mediated by P2X_7_ receptors; bactericidal activity is markedly reduced in P2X_7_ receptor–deficient macrophages [34]. It involves activation of phospholipase D; thus, phospholipase D blockers inhibit killing of intracellular mycobacteria following ATP treatment [34]. Blocking macrophage phospholipase D activity, however, does not inhibit macrophage apoptotic death, demonstrating that, while ATP stimulation leads to macrophage apoptosis and mycobacterial death, these processes can be uncoupled [34]. More recently, autophagy has been shown to have a role in the control of mycobacterial infections. ATP treatment rapidly induces autophagy and mycobacterial killing in a process dependent on P2X_7_ receptor activation and Ca^2+^ influx [35].

Evidence for P2X_7_ receptor involvement in mycobacterial killing comes from studies showing that loss-of-function polymorphisms in the human P2X_7_ receptor gene lead to increased susceptibility to *M. tuberculosis.* Fernando et al. [36] investigated the prevalence of the 1513 A>C polymorphism in two independent Southeast Asian cohorts, and found a strong association with the 1513 A>C polymorphism and extrapulmonary tuberculosis. Subsequent studies have also found that the 1513C allele is a risk factor in the development of extrapulmonary and pulmonary tuberculosis in numerous ethnic populations, including Mexican [37], Russian Slavic [38], and a North Indian Punjabi population [39]. Recently, an association with the 1513C allele and the development of extrapulmonary tuberculosis in Turkish children was also identified [40]. Additional studies have shown associations between tuberculosis and alleles other than 1513C. Li et al. [41] studied various P2X_7_ receptor polymorphisms in a Gambian population. They found that a protective effect against tuberculosis was associated with a P2X_7_ receptor promoter polymorphism at position −762, but the 1513 A>C polymorphism discussed above did not show any significant association. Interestingly, Sambasivan et al. [42] found an association between development of clinical tuberculosis and the presence of the −762C or 1729T allele but not the 1513C allele in an Indian cohort, while Xiao et al. [43] did not find an association between either the 1513C allele or the −762C allele and the development of pulmonary tuberculosis in a Chinese Han cohort. Overall, though, a recent meta-analysis of P2X_7_ receptor gene polymorphism association studies revealed a strong association between the 1513 A>C polymorphism and susceptibility to tuberculosis [44].

Data from in vitro studies provide evidence for potential mechanistic explanations for the association of polymorphisms in the human P2X_7_ receptor gene with susceptibility to tuberculosis. Saunders et al. [45] activated P2X_7_ receptors on BCG-infected macrophages from wild-type and homozygous 1513C donors and showed that P2X_7_ receptor–activated wild-type macrophages undergo apoptosis and kill intracellular bacteria; however, macrophages homozygous for the 1513 A>C loss-of-function polymorphism fail to undergo apoptosis upon exposure to ATP, resulting in mycobacterial survival. The effect of other P2X_7_ receptor polymorphisms was further assessed by Fernando et al. [46] and Shemon et al. [25], who showed that several P2X_7_ receptor polymorphisms (946 G>A, 1729 T>A, and 155+1 g>t) result in reduced macrophage apoptosis and mycobacterial killing. This effect was augmented in compound heterozygous donors (donors with a heterozygous loss of function polymorphism at more than one position on the P2X_7_ receptor gene).

The P2X_7_ receptor has also been implicated in the innate response to obligate intracellular bacteria of the *Chlamydia* genus. The macrophage is an important reservoir and source of dissemination of *Chlamydia* [47], [48]. Like mycobacteria, *Chlamydia* within macrophages are susceptible to ATP treatment, which results in the death of ∼70%–90% of all intracellular bacteria [49]. This appears to be the result of P2X_7_ receptor–dependent stimulation of phospholipase D activity and subsequent phagolysosome fusion. Macrophages from P2X_7_ receptor gene knockout mice show no phospholipase D activation after treatment with ATP and are completely unable to induce ATP-dependent chlamydial death [50]. Furthermore, inhibiting phospholipase D activation in normal murine macrophages, by the addition of butan-1-ol, restores the levels of infection to ∼50%, indicating that phospholipase D is at least partly responsible for chlamydial death [50]. Moreover, treatment of epithelial cells (the preferred target cell for chlamydiae) with P2X_7_ receptor agonists reduces the infectiveness of the bacteria, again associated with activation of phospholipase D [51].

Although *Chlamydia* is clearly vulnerable to P2X_7_ receptor–dependent killing, it has developed some resistance to this killing. *Chlamydia*-infected J774 murine macrophages are resistant to apoptosis following treatment with ATP, whereas uninfected cells undergo apoptosis via P2X_7_ receptor–dependent pathways [49]. Infection results in markedly reduced activation of the P2X_7_ receptor; the mechanism by which this is achieved remains to be elucidated, but it is known that live, growing organisms are required [49].

## The P2X_7_ Receptor and Intracellular Parasites

The P2X_7_ receptor may also play a role in clearance of intracellular parasites of the *Leishmania* genus. Murine macrophages and cells cultured from cutaneous lesions of mice infected with *Leishmania amazonensis* are more sensitive to P2X_7_ receptor–mediated pore formation, inhibiting growth of the intracellular parasite [52]. Killing of *L. amazonensis* via the P2X_7_ receptor is independent of nitric oxide; instead, it is associated with host cell apoptosis [52]. However, when P2X_7_ receptor gene knockout mice and parental C57BL/6J mice are infected intradermally with *Leishmania major*, no difference in resolution of lesions is observed, suggesting that, in the absence of P2X_7_ receptors, other anti-parasitic defence mechanisms compensate to control infection in vivo (C. Miller, A. Zakrzewski, M. Katrib, N. Smith, unpublished observations).

Recently, the P2X_7_ receptor has been implicated in the immune response to another intracellular protozoan, *Toxoplasma gondii*, a parasite that is able to infect and survive in cells of the monocyte/macrophage lineage. However, the role of the P2X_7_ receptor in the response to *T. gondii* is an intriguing and complex one. Activation of “wild-type” human or murine macrophages with ATP induces killing of tachyzoites of both virulent and avirulent strains of the parasite, but the same treatment of macrophages from humans with the 1513 A>C loss-of-function polymorphism or macrophages from P2X_7_ receptor knockout mice has no effect on parasite viability [53], [54]. Interestingly, P2X_7_ receptor–mediated killing of *T. gondii* is not linked to generation of nitric oxide but is, rather, associated with either phagolysosome formation accompanied by production of free oxygen radicals [53] or host cell apoptosis [54]. Thus, P2X_7_ receptor–mediated killing of *T. gondii* has much in common with similarly mediated killing of *Mycobacteria*, *Chlamydia*, and *Leishmania.*

However, susceptibility to either congenital or ocular toxoplasmosis in humans is not associated significantly with the 1513 A>C loss-of-function polymorphism [55]. Intriguingly, though, resistance to both congenital and ocular toxoplasmosis, in the United States and Brazil, respectively, is associated positively with the 1068 T>C polymorphism [55]. This polymorphism has recently been revealed to code for a gain-of-function phenotype in P2X_7_ receptor activity [27] and may be the true ancestral primate sequence [55]. Thus, the American and Brazilian association studies could be interpreted to indicate that reduced inflammatory disease of the foetus or the eye is a consequence of efficient control of parasite load in the presence of a fully functional, ancestral P2X_7_ receptor [55]. Larger scale association studies may be needed to truly resolve the association, or otherwise, of various polymorphisms in the P2X_7_ receptor and congenital or acquired toxoplasmosis.

In vivo murine evidence for a significant role of the P2X_7_ receptor in controlling *T. gondii* is also inconsistent. In one study, where mice were infected intraperitoneally with tachyzoites of the type 2, avirulent ME49 strain of *T. gondii*, splenic parasite burdens in different mouse strains were in proportions consistent with their relative P2X_7_ receptor activity [54]; thus, P2X_7_ receptor knockout mice harboured more parasites than the parental C57BL/6J mice, whereas, in another study, using the same parasites and route of infection, no difference in parasite burden between receptor knockout mice and the parental strain was observed in vivo [56]. C57BL/6J mice are known to possess a proline-to-leucine polymorphism at amino acid 453 in their P2X_7_ receptor that affects some [22], [57]–[59], but not all [22], [60], [61], functions of the receptor and so, C57BL/6J mice, in turn, had higher parasite burdens than resistant BALB/c mice with fully functioning P2X_7_ receptors. It should be noted, however, that BALB/c and C57BL/6J mice display differing susceptibilities to *T. gondii* infection and multiple genes are associated with this [62]. This may help explain the discrepancy seen in the in vivo studies.

The apparent inconsistency between P2X_7_ receptor–mediated killing of *T. gondii* in vitro versus in vivo may simply reflect the fact that type 2 strains of *T. gondii*, like ME49, induce a wide variety of pro-inflammatory responses that can control the reproduction of this parasite [63]. Put another way, P2X_7_ receptor–mediated killing is just one means of control of *T. gondii*, and others remain operative in knockout mice so that little effect on parasite burden is observed in vivo. This does, however, indicate that the P2X_7_ receptor plays a contributory rather than major role in controlling *T. gondii*, which is underscored by the fact that production of several cytokines known to be important in the control of *T. gondii*, including gamma-interferon and IL-12, are not inhibited in *T. gondii*–infected P2X_7_ receptor knockout mice [56].

Most intriguingly, P2X_7_ receptor knockout mice are very susceptible to acute toxoplasmosis, losing weight faster and more dramatically than either C57BL/6J or BALB/c mice [56]. This susceptibility is not related to relative parasite burdens or a general alteration in cytokine responses but is associated with an inability of the knockout mice to limit nitric oxide production [56], a well-known cause of immunopathology in parasitic infections, including *T. gondii* [63]. P2X_7_ receptor knockout mice infected with *T. gondii* also display a delayed—but not abrogated—production of IL-10 [56], which may partially explain their inability to control nitric oxide production. However, it must be stressed that these observations are correlative rather than definitive evidence that the susceptibility to toxoplasmosis apparent in P2X_7_ receptor–deficient mice is due to an over-exuberant inflammatory response and, as this immunoregulatory role for the P2X_7_ receptor has not been noted previously, it does require in-depth follow-up.

It is tempting to speculate that extracellular ATP is an effective danger signal for the immune system, produced as a result of damage caused by factors like nitric oxide. Since nitric oxide is potentially so damaging, its production is tightly controlled, possibly through a negative feedback mechanism. In this scenario, perhaps cells lacking the P2X_7_ receptor to sense extracellular ATP continue producing nitric oxide, whereas cells with functional receptors are able to regulate nitric oxide production. Figure 1 presents a possible molecular explanation. CREB [64] is a transcription factor that has been linked to the suppression of inducible nitric oxide synthase. Extracellular ATP induces activation of CREB as well as suppressing LPS-induced nitric oxide production [65]. Although the exact mechanism has not been elucidated, it is thought that activated CREB sequesters a transcriptional co-activator, CBP, preventing it from interacting with the nuclear factor kappa B (NFκB) subunit p65, thus inhibiting expression of inducible nitric oxide synthase [65]. Hence, during infection with *T. gondii*, extracellular ATP, operating through the P2X_7_ receptor, may activate CREB and down-regulate nitric oxide production. Cells lacking the P2X_7_ receptor would, therefore, be less responsive to any build-up in extracellular ATP. The downstream effect of this would be a lack of phosphorylation, allowing CBP to remain bound to NFκB, and inducible nitric oxide synthase transcription and nitric oxide production to continue indefinitely. Pathology would result.

## Conclusion

Clearly, the P2X_7_ receptor comprises an important part of the host arsenal against invading pathogens. However, there is growing evidence of bacteria and parasites subverting the P2X_7_ receptor pathways for their own advantage, be that through P2X_7_ receptor–mediated apoptosis, phospholipase D production, or inhibition of these, as highlighted above.

We speculate that many other pathogens also modulate the host P2X_7_ receptor. For example, intracellular parasites of various genera—including *Theileria* [66], *Leishmania* [67], [68], *Cryptosporidium* [69], [70], and *Microsporidia* [71], and bacteria such as *Brucella* [72], [73]—all prevent apoptosis of host cells to aid their own survival. Other pathogens, such as *Plasmodium* [74], [75], *Tritrichomonas* [76], [77], *Streptococcus* [78], [79], and *Legionella* [80], actually increase apoptosis of host cells, thereby reducing the numbers of circulating immune cells and increasing their chances for survival. Intriguingly, *T. gondii* is able to both induce and suppress apoptosis in a cell type–dependent manner that allows it to establish a persistent infection [81]. Could some, or all of these, be affecting host cell apotosis via the P2X_7_ receptor? *Legionella* [82], *Francisella* [83], [84], and *Leishmania* [85] prevent phagolysosome fusion—perhaps this is in a P2X_7_ receptor–dependent manner analogous to *Chlamydia*? And, although no definitive link with the P2X_7_ receptor exists, infection of peritoneal macrophages with *Trypanosoma cruzi* down-regulates expression of P2X_7_ receptors [86], which may reduce the immune pressure on these parasites. It would be interesting to investigate whether the P2X_7_ receptor is a common target in the immune evasion strategies of these diverse pathogens. It should also be noted that many of these studies have been conducted in vitro, and it will be important to confirm that the P2X_7_ receptor is playing a non-redundant role in controlling these pathogens in vivo using P2X_7_ receptor knockout mice.

Perhaps surprisingly, extracellular pathogens may also be affected by, or react to, P2X_7_ receptor activity. For example, *Pseudomonas aeruginosa*, a major pathogen in the lungs of cystic fibrosis patients, and *Vibrio cholerae*, the causative agent of cholera, have been shown to secrete multiple enzymes with ATP-modifying activities, such as adenylate kinase, ATPase, and 5′-nucleotidase [87]–[89]. It is believed that these enzymes are used to modulate external ATP levels and, thereby, increase P2X_7_ receptor function, mediating apoptosis of macrophages. Extracellular bacteria, such as *Staphylococcus aureus* and *Escherichia coli*, have been linked to P2X_7_ receptor dependence in a study of caspase-1 activation and subsequent IL-1β secretion [90]. It is unclear in these studies, however, how this stimulation of apoptosis affects the survival and growth of the bacteria. Thus, future work may well reveal that the P2X_7_ receptor has—sometimes quite unexpected—roles in modulating a variety of infectious diseases, not just those that parasitise macrophages.
